# Mendelian Randomization and Single-Cell Transcriptomic Analyses Implicate PPP1R1B as a Risk Factor of Intrahepatic Cholangiocarcinoma

**DOI:** 10.3390/genes17080868

**Published:** 2026-07-25

**Authors:** Zhuomiaoyu Chen, Fumei Cao, Zhao Li

**Affiliations:** Department of Hepatobiliary Surgery, Peking University People’s Hospital, Beijing 100044, China; chenzhmy@163.com (Z.C.); caofm95@163.com (F.C.)

**Keywords:** intrahepatic cholangiocarcinoma, *PPP1R1B*, Mendelian randomization, single cell analysis, transcriptome analysis

## Abstract

**Background:** *PPP1R1B* has been implicated in tumor progression in several malignancies; however, population-level genetic evidence supporting its role in intrahepatic cholangiocarcinoma (ICC) remains limited. This study aimed to investigate the association between *PPP1R1B* and ICC risk and prognosis using genetic and multi-omics analyses. **Methods:** Multiple public datasets were integrated, including finn-b-C3_LIVER_INTRAHEPATIC_BILE_DUCTS, ebi-a-GCST90018583, eqtl-a-ENSG00000131771, GSE138709, The Cancer Genome Atlas (TCGA)-ICC, and FU-iCCA. Two-sample Mendelian randomization (MR) was performed to evaluate the genetic association between *PPP1R1B* expression and ICC risk. *PPP1R1B* expression patterns were further characterized using single-cell and bulk transcriptomic analyses. Expression validation was conducted using TCGA-ICC data and tissue microarray immunohistochemistry. Survival analyses were performed using the FU-iCCA cohort and institutional clinical samples. **Results:** MR analysis identified *PPP1R1B* as significantly associated with increased ICC risk (*p* < 0.05, OR > 1), and sensitivity analyses supported the robustness of the results. Single-cell analysis demonstrated enrichment of PPP1R1B expression in malignant cell populations. Bulk transcriptomic datasets and immunohistochemical validation confirmed elevated PPP1R1B expression in tumor tissues. High PPP1R1B expression was significantly associated with poorer overall survival. **Conclusions:** Genetic and multi-omics evidence supports PPP1R1B as a risk-associated and potential prognostic biomarker in ICC, providing a translational genomics framework for future biomarker development. These findings complement previously reported functional studies and strengthen the biological relevance of *PPP1R1B* in ICC.

## 1. Introduction

Intrahepatic cholangiocarcinoma (ICC) is a highly aggressive form of primary liver cancer (PLC), characterized by insidious onset and rapid progression. Among primary malignant liver tumors, ICC ranks second only to hepatocellular carcinoma, accounting for 10–15% of PLC cases [1]. Globally, the incidence of ICC has been rising significantly, with Asian populations exhibiting a markedly higher incidence than those in Europe and the Americas [2]. The development of ICC is associated with various risk factors, including viral hepatitis, hepatolithiasis, primary sclerosing cholangitis, and intraductal papilloma [3]. Its initiation and progression are closely linked to dysregulation of the cell cycle and evasion of apoptosis [4]. In its early stages, ICC is often asymptomatic. Diagnostic approaches such as enhanced Computed Tomography (CT), Magnetic Resonance Imaging (MRI), and tumor markers like CA199 are frequently used in the auxiliary diagnosis of ICC. However, histopathological examination remains the gold standard for definitive diagnosis. Radical surgical resection is currently the only potentially curative treatment for ICC, though the majority of patients are diagnosed too late to undergo surgery. The five-year survival rate for ICC patients is approximately 30%, with recurrence rates following surgery ranging from 60% to 70% [5]. Given its high malignancy and poor prognosis, the survival outlook for ICC patients remains grim, and effective therapeutic options are scarce. In recent years, there has been growing interest in the molecular mechanisms, progression, and subtypes of ICC. However, the clinical lack of reliable diagnostic markers and therapeutic targets highlights the pressing need for identifying prognostic genes and novel molecular factors, which are crucial for improving patient outcomes.

The *PPP1R1B* gene is located on chromosome 17q12 (39,626,208–39,636,626 [GRCh38/hg38]) and encodes two proteins, Darpp-32 and t-Darpp. It plays a key role in the cAMP signaling pathway, contributing to various biological functions such as bile secretion [6], intracellular signaling, and modulation of several cellular processes, including cell cycle progression and apoptosis [7]. The phosphorylation profile of Darpp-32 regulates its ability to bind and inhibit Protein Phosphatase 1 activity [8]. While early studies suggested that the transcripts and proteins encoded by *PPP1R1B* primarily act on neurons, DARPP-32 plays a pivotal role in downstream signaling mediated by the dopamine D1 receptor subtype, while being negatively regulated through both dopamine D2 receptors and N-methyl-D-aspartate receptors (NMDARs) [9]. Cyclic nucleotide signaling pathways regulate cAMP response element-binding protein (CREB) and DARPP-32, thereby contributing to physiological processes such as synaptic transmission, neuronal excitability, neural plasticity, and neuroprotection [10]. The second protein product, t-Darpp (truncated Darpp-32), was discovered in gastric cancers in 2002 [11]. T-Darpp is a calcium-binding protein that activates PKA through phosphorylation of CDK1 or CDK5 at T39. Since then, overexpression of both Darpp-32 and t-Darpp has been observed in various cancers, including breast, colon, esophageal, gastric, lung, and prostate cancers. Moreover, studies have shown that the invasion and metastasis of pancreatic cancer are inhibited following *PPP1R1B* knockout in pancreatic cancer cell lines and mouse models [7]. Given its functional role, overexpression in multiple malignancies, and involvement in regulating invasion and metastasis in pancreatic cancer, it is hypothesized that *PPP1R1B* may contribute to ICC development through similar mechanisms. However, its precise role in ICC remains unclear. This study, therefore, aims to elucidate the potential mechanisms linking *PPP1R1B* to ICC, providing novel molecular targets and insights for prognostic assessment and targeted therapy.

Mendelian randomization (MR) is a natural experimental design based on genetic variation, used to assess causal relationships between observed biomarkers or environmental factors and specific outcomes, such as disease risk. Rooted in Mendelian genetics, MR leverages the random distribution of genetic variation among individuals to simulate the effects of randomized controlled trials, offering more reliable causal inferences [12]. Recently, MR has gained widespread use in observational research. The discovery of genetic variations strongly associated with specific traits and the availability of large sample genome-wide association studies (GWAS), which have publicly released vast datasets linking exposure, disease, and genetic variation, have enhanced the effectiveness of MR research [13]. Recent MR studies have identified gallstones and liver fat accumulation as risk factors for cholangiocarcinoma [14]. However, there is currently limited research on the relationship between ICC and *PPP1R1B* through MR.

Previous studies have demonstrated through in vitro and in vivo functional experiments that *PPP1R1B* promotes ICC cell proliferation, migration, and invasion [15]. Multiple regression analysis of GWAS data identified *PPP1R1B* as an ICC risk factor. Integrated single-cell RNA sequencing (scRNA-seq) and bulk RNA-seq analyses demonstrated significant PPP1R1B upregulation in ICC tumor cells compared to normal bile duct and liver tissues, and linked high expression to poor prognosis. Investigating this previously unexplored role, the study delineated PPP1R1B-mediated mechanisms in ICC pathogenesis and established PPP1R1B as a risk-associated molecular biomarker and therapeutic target to improve diagnostic precision, treatment efficacy, and prognostic evaluation, offering considerable scientific and translational value. Although functional roles of *PPP1R1B* in ICC have been reported previously, population-level genetic evidence supporting its association with ICC risk remains limited. Therefore, we performed Mendelian randomization and multi-omics analyses to address this knowledge gap.

## 2. Materials and Methods

### 2.1. Tissue Microarray, Immunohistochemistry

Paraffin-embedded tumor and peri-tumor normal liver tissue samples were collected from 59 ICC patients who underwent primary curative resection at the Department of Hepatobiliary Surgery, Peking University People’s Hospital, between 1 January 2021 and 31 December 2023. A total of 29 male and 30 female patients were included. The mean age was 60.627 years with a standard deviation of 10.851. Two normal liver tissue samples were collected from non-tumorous liver tissues in patients who underwent partial hepatectomy for hepatic hemangioma. Ethical approval for the study was granted by the Ethical Review Committee of Peking University People’s Hospital (2023PHB224-001). All tissue samples were obtained from our institution’s tissue bank. All cases were pathologically diagnosed as ICC. Post-operative follow-up was conducted regularly by specialized personnel; patients who could not be contacted after three consecutive attempts were considered lost to follow-up. The final follow-up occurred in April 2024. All patient data were obtained from the electronic medical records and the follow-up database. Tissue microarrays were processed by baking at 60 °C for 1 h, dewaxing in xylene, rehydrating through a graded alcohol series, and blocking endogenous peroxidase activity with 3% hydrogen peroxide. Sections were incubated with 10% goat serum for 30 min to block nonspecific binding, followed by overnight incubation at 4 °C with primary antibody against PPP1R1B (Abcam, ab40801, Cambridge, UK). After extensive washing, sections were incubated with goat anti-mouse or goat anti-rabbit secondary antibody (Vector Lab, CA, Newark, California, USA) at room temperature. Visualization was achieved using the DAB solution, followed by counterstaining with hematoxylin. PPP1R1B expression levels were analyzed using ImageJ software (Version: 2.16.0/1.54p).

### 2.2. Data Extraction

The original MR analysis data for this study were sourced from the Integrative Epidemiology Unit (IEU) Open GWAS database (https://gwas.mrcieu.ac.uk/, accessed on 13 January 2026). The GWAS dataset for ICC (finn-b-C3_LIVER_INTRAHEPATIC_BILE_DUCTS) contained 1,638,446 SNPs from 218,792 samples, serving as the training set. A validation set (ebi-a-GCST90018583) included 1,245,974 SNPs from 159,619 samples. Both outcome datasets employed equivalent ICD-O-3 topography and morphology codes to ensure case purity and exclude mixed hepatobiliary malignancies. The PPP1R1B GWAS dataset (eqtl-a-ENSG00000131771) comprised 17,127 SNPs from 30,645 samples. A single-cell ICC dataset (GSE138709) was downloaded from the Gene Expression Omnibus (GEO) database (https://www.ncbi.nlm.nih.gov/gds, accessed on 13 January 2026), which included 5 ICC tumor tissue samples and 3 controls. The TCGA-ICC dataset, containing 32 ICC samples and 8 control samples with survival data, was obtained from The Cancer Genome Atlas (TCGA) database (https://portal.gdc.cancer.gov/, accessed on 13 January 2026). Additionally, the FU-iCCA dataset, which includes 255 ICC samples with survival data, was sourced from previous studies [16].

### 2.3. MR Analysis

The MR approach is predicated on three core assumptions: (1) the genetic variants are robustly and directly associated with the exposure of interest; (2) these variants are independent of any confounding variables that could bias the exposure–outcome relationship; and (3) the variants influence the outcome solely through the exposure, without exerting pleiotropic effects on other traits.

Instrumental variables (IVs) were identified using the extract_instruments function, which extracts exposure-associated variants surpassing genome-wide significance (*p* < 5 × 10^−8^) [17]. To account for linkage disequilibrium (LD), clumping was performed with parameters set to r^2^ = 0.001 and a window size of 10 kb (clump = TRUE). IVs with F-statistics below 10 were excluded to reduce the risk of weak instrument bias. The F-statistic was calculated using the following formula:

$$F\;=\;\frac{(n-2)\;\times \;r^{2}}{1-r^{2}}$$

Sample size from the GWAS was incorporated into the calculation of the F-statistic, while r^2^ quantified the proportion of variance in the exposure explained by each IV. The extract_outcome_data function was subsequently applied to exclude SNPs unrelated to ICC. Exposure factors associated with fewer than three SNPs were deemed invalid and removed from further analysis. MR analyses were conducted using multiple methods: MR Egger [18,19], weighted median [20], inverse-variance weighted (IVW) [21], simple mode, and weighted mode [22]. Emphasis was placed on results from the IVW method, particularly the *p*-value and odds ratio (OR). A *p*-value < 0.05 was interpreted as evidence of a statistically significant causal relationship between the exposure and outcome. An OR > 1 suggested the exposure acted as a risk factor, whereas an OR < 1 indicated a protective effect.

To evaluate the robustness of the MR analysis, heterogeneity, horizontal pleiotropy, and Leave-One-Out (LOO) sensitivity assessments were performed using the mr_heterogeneity, mr_pleiotropy_test, and mr_leaveoneout functions from the TwoSampleMR package. Cochran’s Q test was applied to examine heterogeneity across SNPs; a Q *p*-value exceeding 0.05 was interpreted as an absence of significant heterogeneity. Horizontal pleiotropy was assessed to detect potential confounding between exposure and outcome datasets, with *p*-values greater than 0.05 suggesting no evidence of directional pleiotropy. The LOO analysis was utilized to identify individual SNPs exerting disproportionate influence on the causal estimates. Any SNP flagged as an outlier during this procedure was excluded from the analysis, and MR estimation was re-conducted to enhance the reliability and interpretability of the causal inference. All analyses adhered to the STROBE-MR reporting guidelines.

### 2.4. Single-Cell Analysis

In the GSE138709 dataset, quality control was performed using the “Seurat” package by excluding cells expressing fewer than 200 genes and genes detected in fewer than 3 cells [23]. Filtering thresholds were set as follows: 300 < nFeature_RNA < 6000, nCount_RNA < 30,000, and percent.mt < 20%. Data normalization was conducted using the NormalizeData function, and highly variable genes were identified *via* FindVariableFeatures, leveraging the mean–variance relationship. Subsequent normalization was executed through the ScaleData function. Dimensionality reduction was achieved using principal component analysis (PCA), followed by cell clustering via uniform manifold approximation and projection (UMAP) with a resolution parameter of 0.5. Cell types were annotated using SingleR with the normalized log-expression matrix from the Seurat object and the Human Primary Cell Atlas (HPCA) as reference. Annotation was performed at the cluster level using a 0.5 quantile threshold for marker gene selection, with resulting labels assigned to clusters and stored in the “Seurat” metadata. The expression profile of PPP1R1B across cell subsets was visualized using the DotPlot function in Seurat. The percentage of PPP1R1B-positive cells was quantified across all annotated cell populations. For pseudotime trajectory inference, the “monocle3” package [24] was employed.

### 2.5. Gene Set Enrichment Analysis (GSEA)

Cells were stratified into high- and low-*PPP1R1B*-expression groups using the median expression value as a threshold. Differential gene expression analysis was performed using the FindMarkers function in “Seurat” (min.pct = 0.1, logfc.threshold = 0, test.use = “wilcox”), and genes were ranked by log fold change (from high to low). GSEA was applied using the “clusterProfiler” package to the ranked list using KEGG pathway gene set (c2.cp.kegg.v7.4.symbols.gmt), adopting *p*.adj < 0.05 and |NES| ≥ 1 as significance criteria.

### 2.6. Transcriptome Analysis

In the TCGA-ICC transcriptomic dataset, *PPP1R1B* expression differences were assessed using the Wilcoxon test. For the FU-iCCA dataset, ICC samples were divided into high- and low-PPP1R1B-expression groups based on the median, and survival outcomes were compared *via* Kaplan–Meier (K-M) analysis. In this study, univariate and multivariate Cox regression analyses were performed on PPP1R1B and age, sex, tumor size, Vascular_invasion, Lymph_node_metastasis, CA199_high, TNM stage (HR ≠ 1, *p* < 0.05).

### 2.7. Statistical Analysis

All bioinformatics analyses were performed using R. Group comparisons utilized the Wilcoxon test, with *p*-values < 0.05 considered statistically significant. Survival differences were evaluated using KM and log-rank tests.

## 3. Results

### 3.1. Ppp1r1b Was a Risk Factor for ICC in MR Analysis

**Figure 1:**
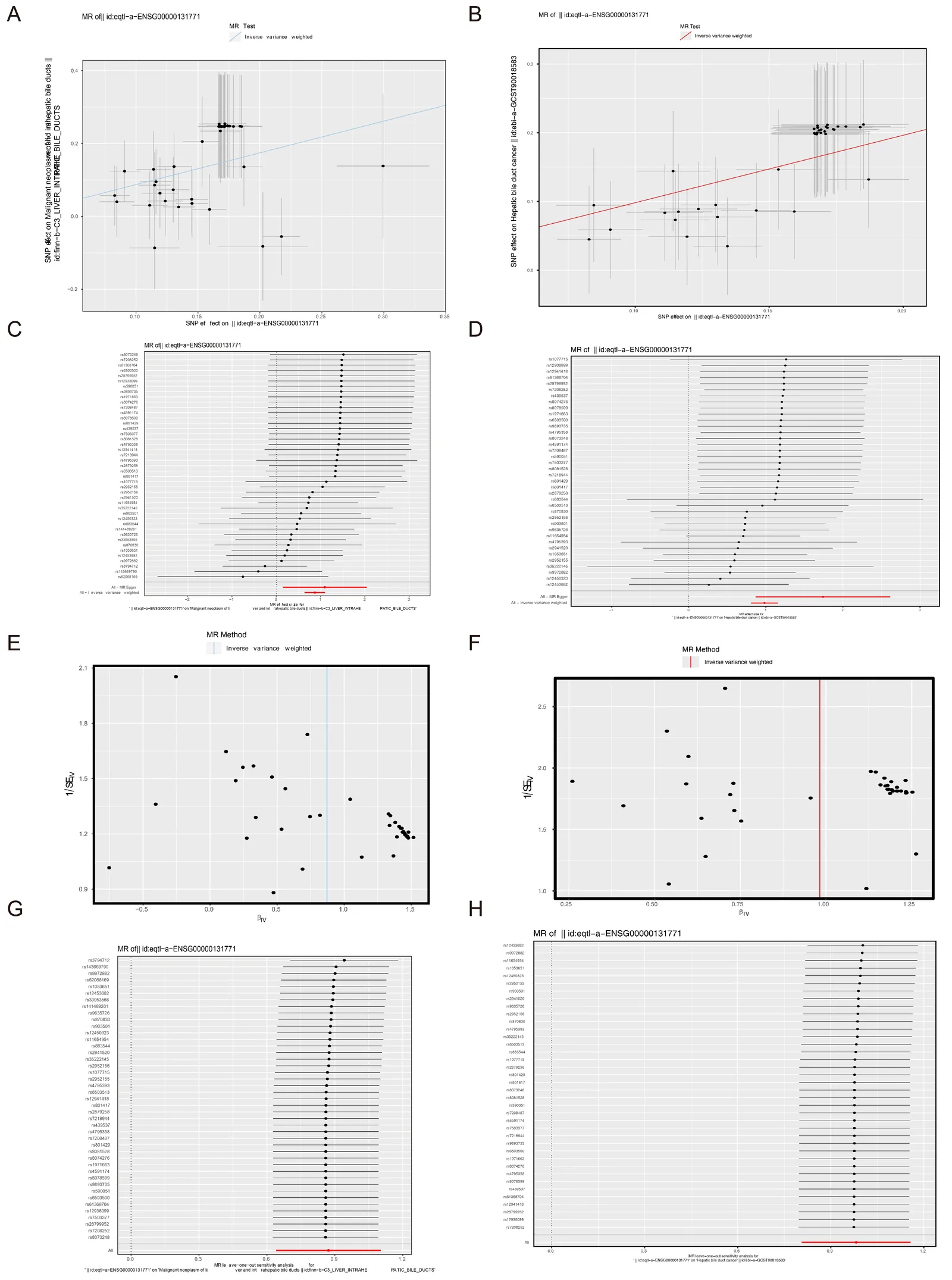
Mendelian randomization (MR) analysis evaluating the association between *PPP1R1B* and intrahepatic cholangiocarcinoma (ICC). (**A**,**B**) Scatter plots illustrating the causal association between *PPP1R1B* and ICC in the (**A**) training set and (**B**) validation set. The slope of each line represents the estimated effect derived from different MR methods. (**C**,**D**) Forest plots showing the effect estimates of *PPP1R1B* on ICC in the (**C**) training set and (**D**) validation set. Red points indicate the pooled estimates obtained using the inverse variance weighted (IVW) method across all single-nucleotide polymorphisms (SNPs), with horizontal lines representing 95% confidence intervals. (**E**,**F**) Funnel plots from reverse MR analysis in the (**E**) training set and (**F**) validation set. Vertical lines indicate overall estimates based on all SNPs. Symmetrical distribution suggests no evidence of horizontal pleiotropy. (**G**,**H**) Leave-one-out sensitivity analysis in the (**G**) training set and (**H**) validation set. Black points indicate the IVW estimates after excluding each individual SNP sequentially. The red point represents the IVW estimate using the complete set of SNPs.

This study aimed to assess the causal relationship between the *PPP1R1B* gene and ICC using MR analysis. Initially, 43 SNPs were identified as being associated with *PPP1R1B* but not with ICC (Supplementary Tables S1–S3). Comprehensive MR results derived from five distinct analytical methods are summarized in Supplementary Table S4. Notably, the IVW method indicated a significant causal association between *PPP1R1B* and ICC (*p* < 0.05), with an OR of 2.391, implicating *PPP1R1B* as a risk factor for ICC. Upon substituting the outcome dataset with the validation set ebi-a-GCST90018583 and repeating the MR analysis, 38 SNPs associated with *PPP1R1B* and unlinked to ICC were selected (Supplementary Tables S1, S5 and S6). Supplementary Table S7 presents multivariate regression outcomes from five MR approaches, among which the IVW method yielded an OR of 2.669 (*p* < 0.05), again exceeding 1 and reinforcing the risk association. Scatter plots from both datasets revealed positive linear trends, corroborating the risk role of PPP1R1B in ICC (Figure 1A,B), while forest plots further supported the association between elevated PPP1R1B expression and increased ICC susceptibility (Figure 1C,D). Additionally, funnel plots demonstrated approximately symmetrical IV distributions around the IVW line, indicating consistency with the assumption of random instrument selection (Figure 1E,F).

Sensitivity analysis was performed to evaluate the robustness of the MR results. Q values exceeding 0.05 in both datasets indicated no significant heterogeneity and demonstrated strong internal consistency (Table 1 and Table 2). Horizontal pleiotropy tests yielded *p* values above 0.05, confirming the absence of significant confounding factors (Table 3), with validation set results corroborating these findings (Table 4), thereby reinforcing result reliability. Sequential SNP exclusion revealed no substantial changes in the effect estimates on outcome variables, confirming the stability of the MR findings (Figure 1G); consistent results were observed in the validation set (Figure 1H), further substantiating the robustness of the analysis. Overall, analyses using ebi-a-GCST90018583 and finn-b-C3_LIVER_INTRAHEPATIC_BILE_DUCTS endpoints displayed high concordance, indicating a direct causal link between *PPP1R1B* expression and ICC risk, with elevated PPP1R1B levels associated with increased ICC susceptibility.

### 3.2. A Total of 19 Different Cell Clusters Were Identified in GSE138709

**Figure 2:**
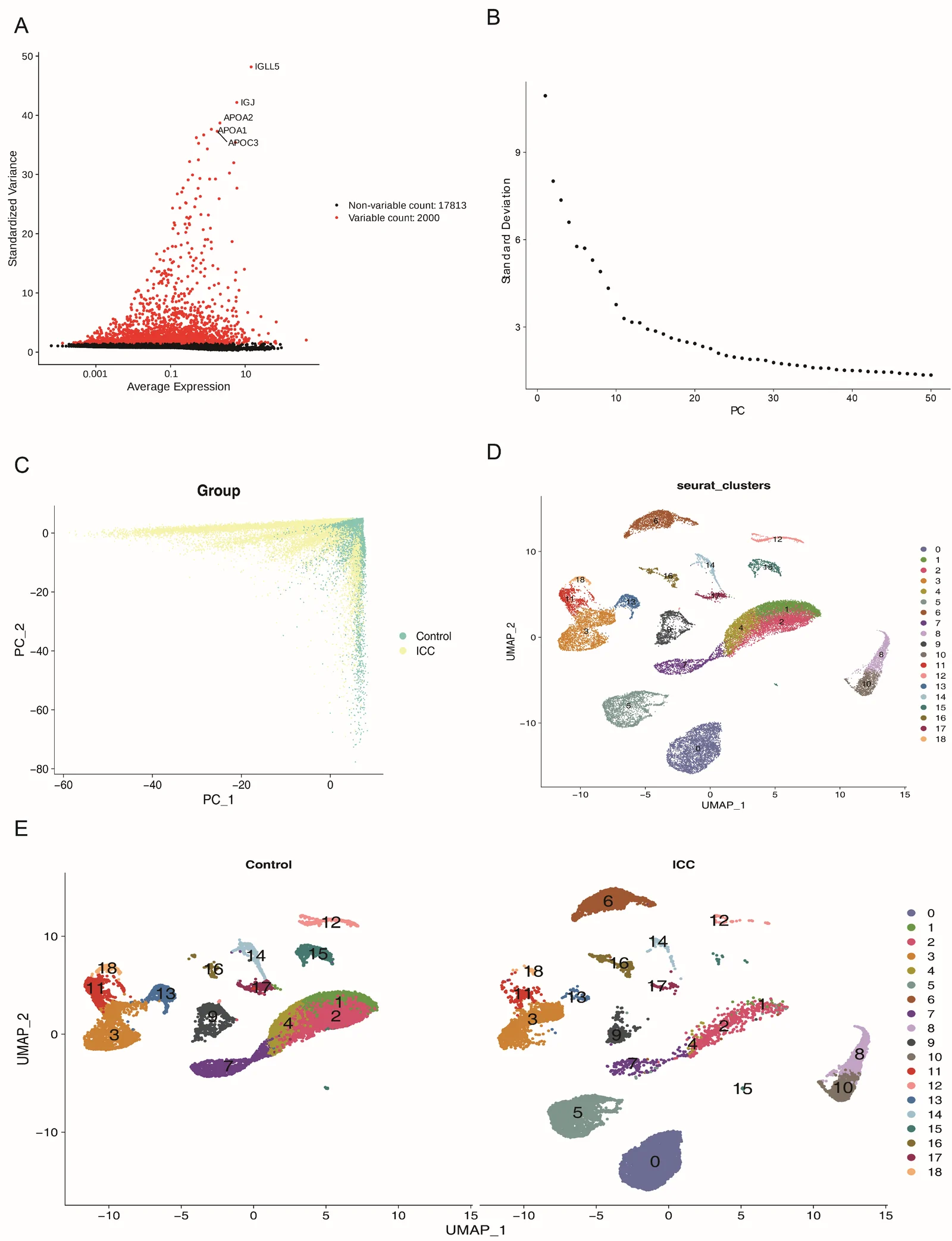
scRNA-seq analysis of intrahepatic cholangiocarcinoma (ICC). (**A**) Identification of 2000 highly variable genes, with the top five highlighted. (**B**) Scree plot depicting the contribution of the first 50 principal components (PCs) to overall variance; the first 20 PCs were selected for downstream analysis. (**C**) PCs of ICC and control samples exhibit relatively high aggregation, indicating distinct data structure. (**D**) Uniform manifold approximation and projection (UMAP) clustering identified 19 distinct cell populations across all samples. (**E**) UMAP clustering results for ICC and control groups, respectively.

The dataset underwent quality control to identify cell clusters and specific clusters associated with the ICC group, resulting in 31,572 cells and 19,813 genes retained in GSE138709 (Supplementary Figure S1A,B). After standard processing, 2000 highly variable genes were selected (Figure 2A). PCA was conducted, and the first 20 principal components (PCs) were chosen for subsequent analysis (Figure 2B,C). At a resolution of 0.5, the data partitioned into 19 distinct clusters that aligned well with established ICC cell types, achieving an optimal balance without over-fragmentation. UMAP clustering identified 19 distinct cell clusters (Figure 2D), with clusters 0, 5, 6, 8, and 10 specifically found in the ICC group (Figure 2E).

### 3.3. Ppp1r1b Was Specifically Expressed in the ICC Group

**Figure 3:**
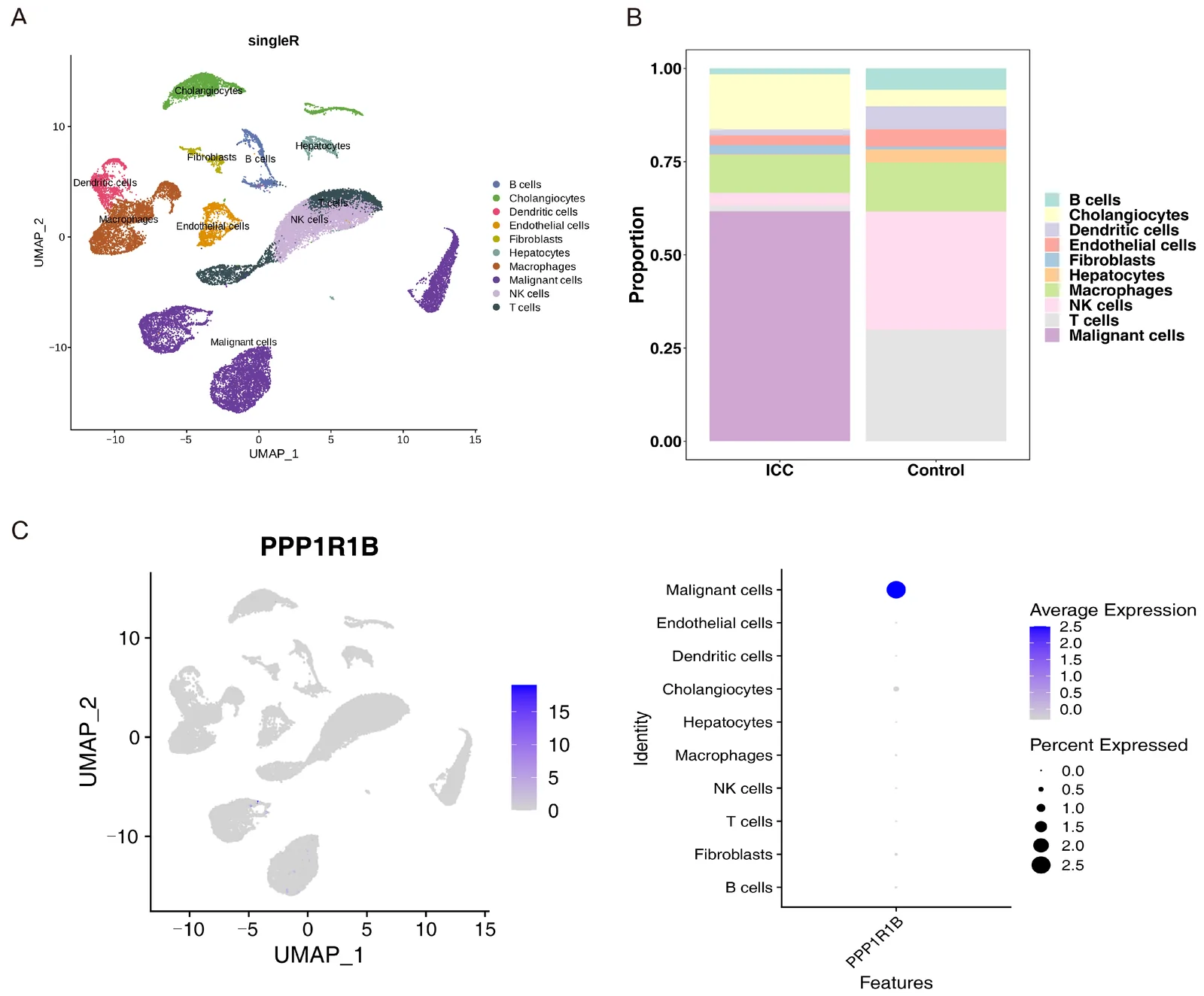
Cell subpopulation classification and PPP1R1B expression analysis. (**A**) Ten cell subpopulations were identified based on canonical marker genes. (**B**) Comparison of subpopulation proportions between ICC and control groups. (**C**) PPP1R1B is predominantly expressed in malignant cells, with minimal expression in other cell types.

Based on SingleR algorithm, these clusters were classified into 10 cell subpopulations: malignant cells, cholangiocytes, hepatocytes, macrophages, dendritic cells, natural killer cells, fibroblasts, T cells, B cells, and endothelial cells (Figure 3A). Malignant cells were distinguished from normal cholangiocytes through a two-step strategy. First, epithelial clusters present exclusively in ICC samples but absent in control samples were preliminarily classified as malignant cells, whereas epithelial clusters consistently detected in control samples were designated as normal cholangiocytes. Second, these classifications were validated using established lineage markers from published ICC scRNA-seq studies: malignant cells were identified by co-expression of EPCAM, KRT19, and KRT7, while normal cholangiocytes were characterized by FYXD2, TM4SF4, and ANXA4 expression. Histograms comparing the proportion of cell subpopulations between groups showed that malignant cells were most abundant in ICC, while natural killer cells and T cells predominated in controls (Figure 3B). PPP1R1B showed preferential expression in malignant cells within the scRNA-seq cohort (Figure 3C), a finding that requires further validation in larger cohorts. PP1R1B was technically detectable in all ICC cells and all ICC malignant cells (100% positivity; Supplementary Figure S3). *PPP1R1B* enrichment in malignant cell subpopulation suggests its potential utility as a complementary indicator of tumor heterogeneity, though its sparse distribution precludes application as a standalone diagnostic or prognostic marker for ICC.

### 3.4. Ppp1r1b Was Enriched in Neurodegenerative Disease-Associated and Oxidative Phosphorylation Pathways

**Figure 4:**
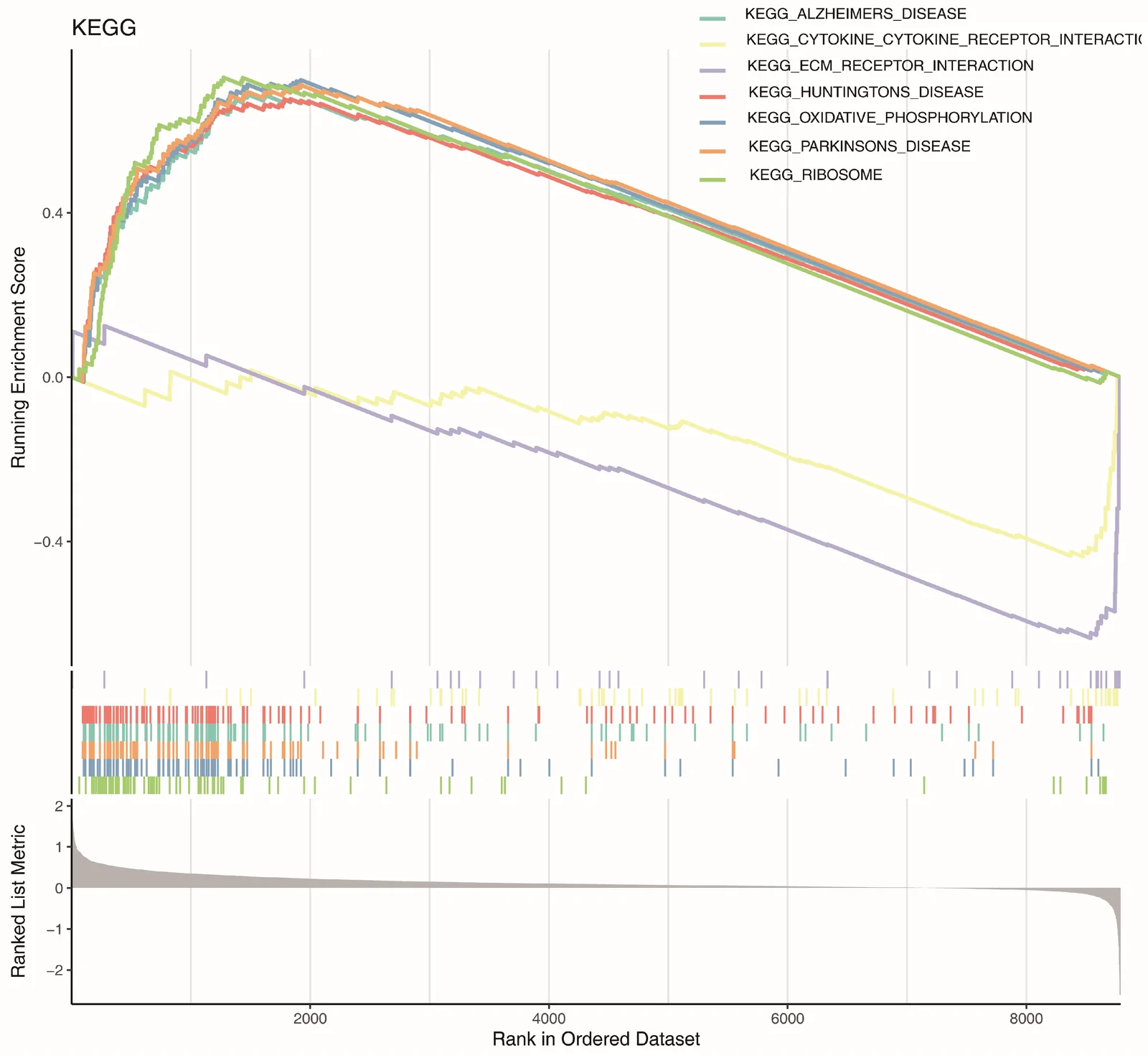
Gene Set Enrichment Analysis (GSEA) of *PPP1R1B* based on KEGG pathways.

To explore *PPP1R1B*’s biological function, GSEA was performed. Its activation pathways were enriched in ribosome, oxidative phosphorylation, Parkinson’s disease, Alzheimer’s disease, and Huntington’s disease, while inhibitory pathways involved cytokine–cytokine receptor and ECM–receptor interactions, which may be key to PPP1R1B’s function (Figure 4). In conclusion, GSEA results provide insight into *PPP1R1B*’s potential biological role.

### 3.5. Ppp1r1b Was Associated with Malignant Cell Differentiation

**Figure 5:**
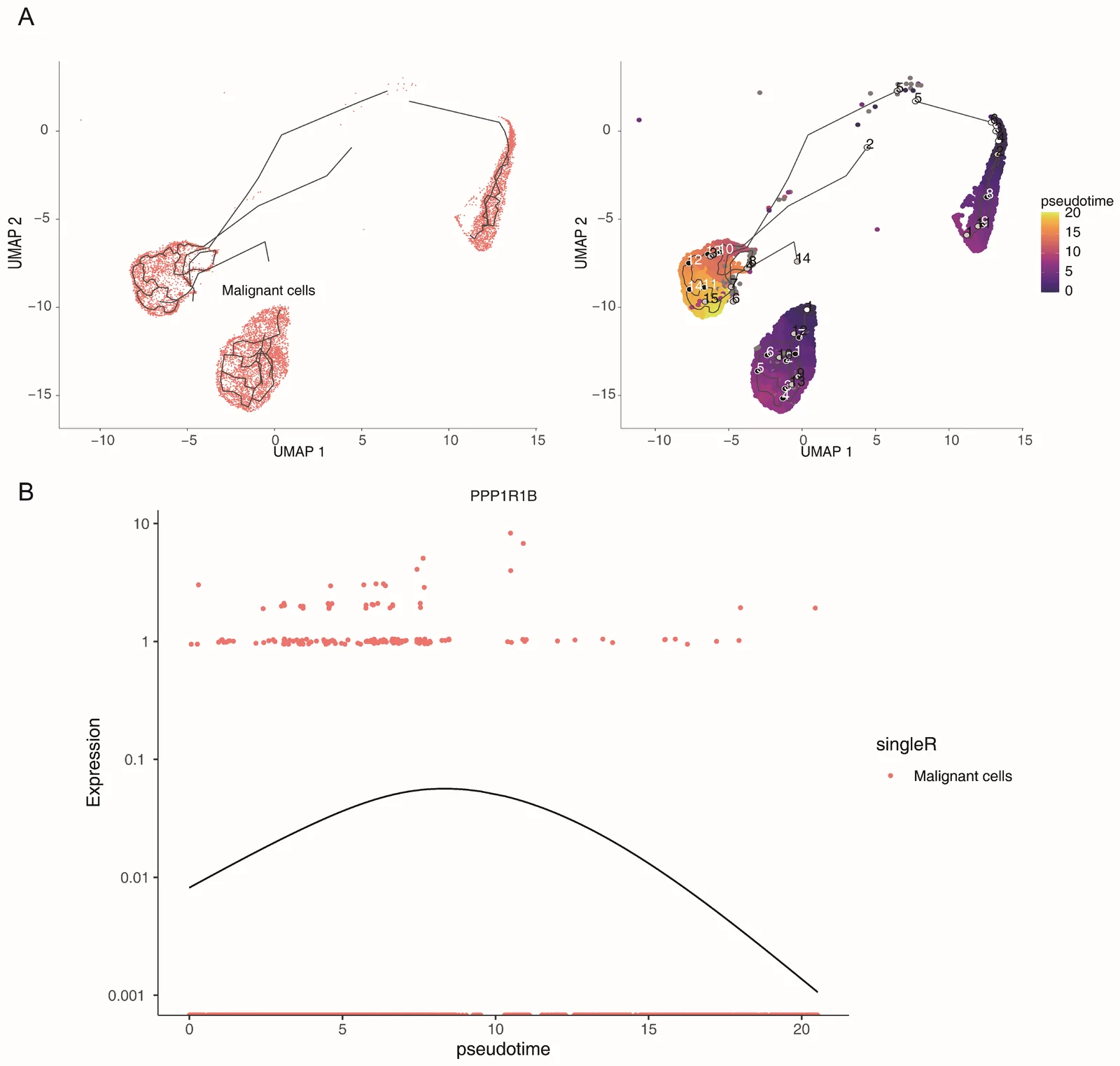
Pseudotime analysis and PPP1R1B expression trajectory. (**A**) Pseudotime inference of malignant cells revealed similar differentiation states among two of the three malignant cell clusters. (**B**) PPP1R1B expression increased initially and subsequently decreased along the pseudotemporal axis.

This analysis aimed to examine the expression pattern of PPP1R1B along the differentiation trajectory of malignant cells (Figure 5A). PPP1R1B expression exhibited a dynamic trend—initially low, increasing to a peak, and subsequently declining (Figure 5B). To delineate distinct differentiation stages, cells were stratified into two groups based on their positions along the pseudotime axis. Cells at the early stage of differentiation were designated as the Low_pseudotime group, whereas those at the later stage comprised the High_pseudotime group. Collectively, the pseudotemporal trajectory analysis revealed a dynamic expression profile of PPP1R1B during malignant cell differentiation. Additionally, in the pseudotime analysis, we did not perform parametric trend fitting; instead, we plotted raw expression values with LOESS-smoothed curves to illustrate the approximate distribution. Given the limited number of PPP1R1B-positive cells, this pattern should be regarded as exploratory and requires validation in larger cohorts.

### 3.6. Results of Transcriptome Analysis and Tissue Microarray (TMA) Immunohistochemistry Were Consistent with Single-Cell Analysis and MR Analysis

**Figure 6:**
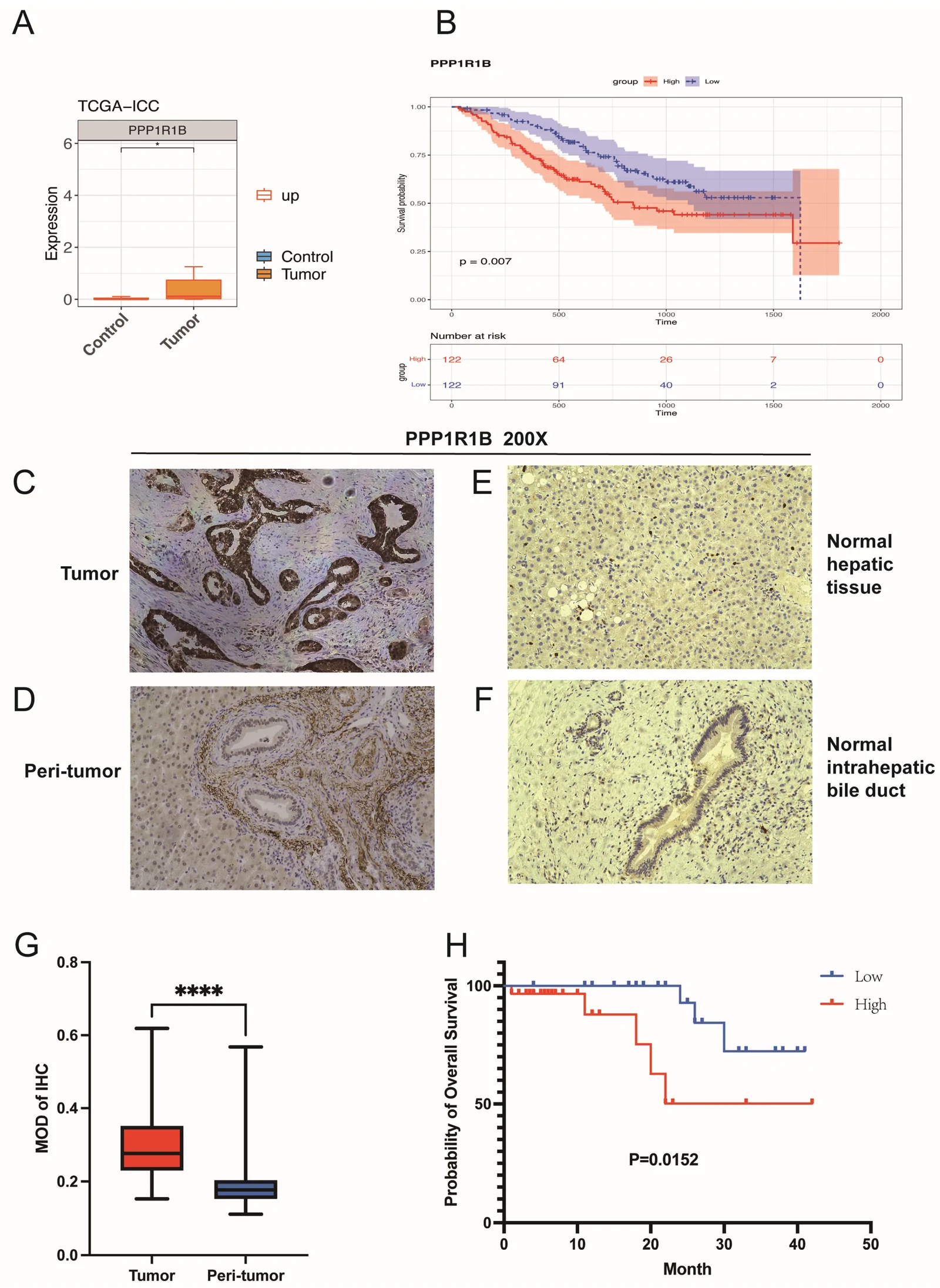
PPP1R1B expression in bulk RNA-seq and survival analysis. (**A**) PPP1R1B expression was significantly higher in ICC tumor tissues compared to adjacent non-tumor tissues. (**B**) Patients with high PPP1R1B expression showed poorer survival in the FU dataset (*p* < 0.05). (**C**–**F**) Expression levels of PPP1R1B in (**C**) ICC tumor tissues, (**D**) peri-tumoral liver, (**E**) normal hepatic tissue, and (**F**) normal intrahepatic bile ducts, respectively. (**G**) Tissue microarray (TMA) immunohistochemical staining confirmed elevated PPP1R1B expression in ICC tumors (**** *p* < 0.0001). (**H**) High PPP1R1B expression was associated with worse prognosis in the independent cohort (*p* < 0.05).

The prognostic relevance of PPP1R1B in ICC was also evaluated. Consistent with its expression pattern in GSE138709, PPP1R1B was markedly upregulated in the ICC cohort of TCGA-ICC (Figure 6A). To assess its prognostic implication, ICC samples in the FU-iCCA dataset were categorized into high- and low-expression groups based on the median PPP1R1B expression level. KM survival analysis indicated that elevated PPP1R1B expression was associated with poorer prognosis, corroborating the MR analysis results (Figure 6B). Univariate analysis confirmed that high PPP1R1B expression was significantly associated with worse overall survival (*p* < 0.05) (Supplementary Table S8). However, after adjusting for all clinical confounders, the prognostic effect of PPP1R1B became marginally significant (*p* = 0.058) (Supplementary Figure S2) and failed to meet the statistical criteria for an independent prognostic factor. This indicates that the survival predictive value of PPP1R1B is confounded by clinicopathological features such as tumor stage and lymph node metastasis, precluding its use as an independent prognostic biomarker in the adjusted model. Immunohistochemical staining of TMA specimens from our department (Figure 6C,D) similarly demonstrated significantly higher PPP1R1B expression in ICC tumor tissues compared to adjacent non-tumor tissues (Figure 6G). In addition, staining of two normal liver tissue samples which collected from non-tumorous liver tissues in patients undergoing partial hepatectomy for hepatic hemangioma revealed substantially lower PPP1R1B expression in both normal hepatic parenchyma and intrahepatic bile ducts relative to ICC tumor tissues (Figure 6E,F). Survival analysis further showed that patients with high PPP1R1B expression had reduced postoperative overall survival compared to those with low expression levels (Figure 6H). Collectively, these findings suggest that PPP1R1B may serve as a potential prognostic indicator in ICC, with elevated expression associated with worse clinical outcome in univariate analysis; however, its prognostic independence is limited after adjusting for established clinicopathological confounders.

## 4. Discussion

ICC is a malignant tumor originating from the epithelial lining of the secondary bile ducts to the smallest intrahepatic branches and the associated peribiliary glands. It expresses cholangiocyte markers and accounts for approximately 20–30% of all cholangiocarcinomas [25]. ICC is characterized by high malignancy, a lack of effective treatments, and generally poor prognosis. Increasing numbers of sequencing studies have identified potential driver genes in ICC, such as TP53, KRAS, and IDH1 [26]. Different mutation types may correspond to distinct etiologies and risk factors; for instance, TP53 mutations are frequently associated with hepatitis B virus infection. The *PPP1R1B* gene primarily encodes two isoforms: Darpp-32 and t-Darpp. Darpp-32, identified earlier, is phosphorylated in response to cAMP signaling in dopamine-responsive brain tissues. This phosphorylation regulates its ability to bind and inhibit protein phosphatase 1, thereby influencing the activity of various phosphorylated proteins. t-Darpp, discovered later, has been shown to promote tumor formation, enhance cell migration, and contribute to drug resistance. It forms a complex with insulin-like growth factor 1 receptor (IGF1R), thereby increasing glucose uptake and catabolism [27], and activates downstream signaling through the AKT anti-apoptotic pathway. With further research, both Darpp-32 and t-Darpp have been implicated in promoting cell proliferation, primarily *via* AKT phosphorylation [28]. In gastric cancer cells, overexpression of Darpp-32 enhances invasive capacity, accompanied by upregulation of membrane-type matrix metalloproteinases (MT-MMPs) and CXC chemokine receptor 4 (CXCR4). CXCR4 knockout reduces Darpp-32-induced cell migration, suggesting a mechanistic link [29]. Although previous studies in other cancers have identified PPP1R1B as a contributor to poor prognosis and increased proliferation, migration, and invasion, its role in ICC remains unreported. To investigate this relationship, MR analysis was conducted, revealing PPP1R1B as a potential risk factor for ICC. This association was validated in an independent cohort with consistent findings, supporting a causal link between elevated PPP1R1B expression and increased ICC risk. Subsequent analyses based on single-cell and bulk RNA sequencing further explored PPP1R1B expression and its correlation with patient survival. Additionally, MR analysis identified certain behavioral factors, such as sleep patterns, as ICC risk modifiers, while specific gut microbiota were associated with reduced ICC risk [30,31].

Analysis of scRNA-seq data revealed that PPP1R1B was highly expressed exclusively in tumor cells, with negligible expression in normal bile duct cells and hepatocytes, suggesting that elevated PPP1R1B expression may contribute to the initiation and progression of ICC. Pseudotime trajectory analysis further demonstrated a dynamic expression pattern, with PPP1R1B levels initially increasing and subsequently decreasing along the tumor cell differentiation trajectory. Moreover, the enrichment of *PPP1R1B* in poorly differentiated tumor cells implicates it in higher tumor grade, increased invasive and metastatic potential, and worse clinical outcomes—an association further supported by follow-up survival analyses. A study of 100 colorectal cancer (CRC) patients found PPP1R1B to be significantly overexpressed in cases with distant metastases, identifying it as a key predictor of metastatic spread and unfavorable prognosis [32]. Under CRC conditions, the PEAK1–PPP1R12B axis has been reported to regulate cell proliferation, with aberrant activation of the Wnt/β-catenin pathway identified as a central pathogenic mechanism [33]. The same pathway, when activated, similarly drives uncontrolled proliferation in intrahepatic cholangiocarcinoma (ICC) [34]. These mechanistic parallels suggest that PPP1R1B may play a similar oncogenic role in ICC. Research in pancreatic cancer models demonstrated that HIF1A depletion led to increased PPP1R1B expression, accompanied by TP53 degradation. Functional knockdown of *PPP1R1B* suppressed tumor cell invasiveness both in vitro and in vivo. Consistently, data from TCGA also showed that high PPP1R1B expression correlated with poorer survival outcomes [7]. Pathway enrichment analysis in this study indicated that *PPP1R1B* activation was associated with oxidative phosphorylation, Parkinson’s disease, Alzheimer’s disease, and Huntington’s disease pathways. These findings align with previous studies and further highlight phosphorylation as a potentially critical mechanism in ICC pathogenesis. Notably, activation of the AKT pathway—previously shown to prolong tumor cell survival and promote chemoresistance *via* phosphorylation—may underlie the oncogenic role of *PPP1R1B* in ICC [35].

This study employed MR, integrated with scRNA-seq, bulk RNA-seq, and GWAS data, to investigate the association between *PPP1R1B* and ICC. The findings identified PPP1R1B as a potential risk factor for ICC. Clinical data analysis further demonstrated that PPP1R1B serves as a predictor of poor prognosis in ICC. The use of MR enabled a more reliable assessment of causality by minimizing residual confounding, thereby enhancing the robustness of the association compared to conventional observational studies or even randomized controlled trials. The elevated expression of PPP1R1B in ICC tumor cells, initially revealed through scRNA-seq analysis, was corroborated by large-cohort RNA-seq data and TMA-based immunohistochemistry.

Despite these findings, several limitations should be acknowledged. First, the present study did not include new in vitro or in vivo functional experiments. However, previous work from our group has already provided biological validation demonstrating that *PPP1R1B* regulates tumor cell proliferation, migration, and invasion in ICC models, and has also explored upstream regulatory pathways [15]. Therefore, the current study was designed to complement these functional observations by providing population-level genetic evidence and multi-omics characterization using Mendelian randomization and transcriptomic analyses. Together, these complementary approaches strengthen the biological plausibility and translational relevance of *PPP1R1B* in ICC.

Second, although the MR framework adhered to core assumptions and utilized rigorously selected instrumental variables, with pleiotropy testing and sensitivity analyses performed, the exclusion-restriction assumption—namely that SNPs influence ICC risk solely through PPP1R1B expression—cannot be empirically proven. Residual confounding and undetected horizontal pleiotropy cannot be completely excluded. Future studies incorporating multivariable MR or additional genetic datasets may further improve causal inference. In addition, the use of whole-blood eQTLs as instrumental variables for PPP1R1B expression in ICC may introduce tissue-mismatch bias. Future MR studies utilizing liver- or cholangiocyte-specific eQTL datasets would strengthen the biological relevance of the causal inference.

Third, the single-cell dataset analyzed in this study included a limited number of samples, which may not fully capture the cellular heterogeneity of ICC. Future studies with larger multi-center cohorts and integrative multi-omics analyses may enhance the robustness and generalizability of these findings.

Fourth, PPP1R1B expression was statistically compared between paired tumor and adjacent non-tumorous tissues from 59 patients with ICC, and two normal liver tissue samples were additionally included as negative controls for illustrative comparison. Nevertheless, the overall sample size remained relatively limited, particularly with respect to histologically normal liver tissues. Future studies involving larger independent cohorts and a greater number of normal liver tissue samples are warranted to validate these findings.

## 5. Conclusions

This study identified *PPP1R1B* as a risk factor for ICC through MR analysis. Integrated analysis of scRNA-seq, bulk RNA-seq, and TMA immunohistochemical staining revealed that PPP1R1B was highly expressed in ICC tumor tissues but exhibited minimal expression in normal tissues. These findings suggest a potential role for PPP1R1B in the initiation and progression of ICC. Pseudotime analysis further demonstrated that PPP1R1B expression was elevated in poorly differentiated ICC compared to well-differentiated subtypes. Additionally, survival analysis indicated that high PPP1R1B expression was associated with worse clinical outcomes, implicating it as a potential marker of tumor aggressiveness and poor prognosis. Collectively, these results highlight PPP1R1B as a novel risk factor for ICC and suggest it may serve as a potential therapeutic target in future interventions.

## Figures and Tables

**Table 1:** Heterogeneity test results of MR study in the training set (finn-b-C3_LIVER_INTRAHEPATIC_BILE_DUCTS).

ID.Exposure	ID.Outcome	Outcome	Exposure	Method	Q	Q_df	Q_pval
eqtl-a-ENSG00000131771	finn-b-C3_LIVER_INTRAHEPATIC_BILE_DUCTS	Malignant neoplasm of liver and intrahepatic bile ducts || id:finn-b-C3_LIVER_INTRAHEPATIC_BILE_DUCTS	|| id:eqtl-a-ENSG00000131771	MR Egger	28.28237624	41	0.934165578
eqtl-a-ENSG00000131771	finn-b-C3_LIVER_INTRAHEPATIC_BILE_DUCTS	Malignant neoplasm of liver and intrahepatic bile ducts || id:finn-b-C3_LIVER_INTRAHEPATIC_BILE_DUCTS	|| id:eqtl-a-ENSG00000131771	Inverse variance weighted	28.51128044	42	0.944374401

**Table 2:** Heterogeneity test results of MR study in the validation set (ebi-a-GCST90018583).

ID.Exposure	Id.Outcome	Outcome	Exposure	Method	Q	Q_df	Q_pval
eqtl-a-ENSG00000131771	ebi-a-GCST90018583	Hepatic bile duct cancer || id:ebi-a-GCST90018583	|| id:eqtl-a-ENSG00000131771	MR Egger	‘7.58882694271342	36	0.999999883631249
eqtl-a-ENSG00000131771	ebi-a-GCST90018583	Hepatic bile duct cancer || id:ebi-a-GCST90018583	|| id:eqtl-a-ENSG00000131771	Inverse variance weighted	‘10.6065614425141	37	0.999993789279401

**Table 3:** Horizontal pleiotropy test results of MR study in the training set (finn-b-C3_LIVER_INTRAHEPATIC_BILE_DUCTS).

ID.Exposure	ID.Outcome	Outcome	Exposure	Egger_ Intercept	SE	pval
eqtl-a-ENSG00000131771	finn-b-C3_LIVER_INTRAHEPATIC_BILE_DUCTS	Malignant neoplasm of liver and intrahepatic bile ducts || id:finn-b-C3_LIVER_INTRAHEPATIC_BILE_DUCTS	|| id:eqtl-a-ENSG00000131771	−0.034452242	0.072009634	0.63487905

**Table 4:** Horizontal pleiotropy test results of MR study in the validation set (ebi-a-GCST90018583).

ID.Exposure	ID.Outcome	Outcome	Exposure	Egger_ Intercept	SE	pval
eqtl-a-ENSG00000131771	ebi-a-GCST90018583	Hepatic bile duct cancer || id:ebi-a-GCST90018583	|| id:eqtl-a-ENSG00000131771	−0.11608154437462	0.0668224913523825	0.0909086916021581

## Data Availability

The original detailed data of Mendelian Randomization (MR) analysis in this study were from the Integrative Epidemiology Unit (IEU) Open GWAS database (https://gwas.mrcieu.ac.uk/, accessed on 13 January 2026). The intrahepatic cholangiocarcinoma (ICC) dataset finn-b-C3_LIVER_INTRAHEPATIC_BILE _DUCTS included 1,6380,446 single-nucleotide polymorphisms (SNPs); ebi-a-GCST90018583 included 1,2451,974 SNPs. The PPP1R1B dataset (eqtl-a-ENSG00000131771) included 17,127 SNPs. A single-cell dataset of ICC (GSE138709) was downloaded from the Gene Expression Omnibus (GEO) database (https://www.ncbi.nlm.nih.gov/gds, accessed on 13 January 2026). The TCGA-ICC dataset of ICC was obtained from the Cancer Genome Atlas (TCGA) database (https://portal.gdc.cancer.gov/, accessed on 13 January 2026).

## Supplementary Materials

The following supporting information can be downloaded at https://www.mdpi.com/article/10.3390/genes17080868/s1. Table S1: Exposure dataset utilized for the selection of instrumental variables. Table S2: Outcome dataset employed to identify instrumental variables (finn-b-C3_LIVER_INTRAHEPATIC_BILE_DUCTS). Table S3: Harmonized dataset used to screen 43 SNPs associated with PPP1R1B but unlinked to ICC (finn-b-C3_LIVER_INTRAHEPATIC_BILE_DUCTS). Table S4: Mendelian randomization (MR) analysis evaluating the causal relationship between PPP1R1B and ICC (finn-b-C3_LIVER_INTRAHEPATIC_BILE_DUCTS). Table S5: Outcome dataset applied for instrumental variable selection (ebi-a-GCST90018583). Table S6: Harmonized dataset used to identify 43 SNPs associated with PPP1R1B and not associated with ICC (ebi-a-GCST90018583). Table S7: MR-based causal inference between PPP1R1B and ICC (ebi-a-GCST90018583). Table S8: Univariate Cox proportional hazards regression analysis of overall survival in the FU-iCCA cohort. Figure S1: Single-cell RNA sequencing (scRNA-seq) analysis and annotated cells identification in ICC. (A) Violin plots depicting nFeature_RNA, nCount_RNA, and percent.mt prior to quality control. (B) Violin plots of the same metrics following quality control. Figure S2: The PPP1R1B positive cell percentage in all ICC cells and ICC malignant cells. Figure S3: Results of multivariate Cox regression analysis.

## Abbreviations

MR: Mendelian Randomization; ICC: intrahepatic cholangiocarcinoma; PLC: primary liver cancer; TMA: tissue microarray; IHC: immunohistochemistry.
